# A 13-Week Repeated Oral Dose Toxicity Study of ChondroT in Sprague-Dawley Rats

**DOI:** 10.1186/s12906-019-2773-4

**Published:** 2019-12-12

**Authors:** Jiwon Jeong, Kiljoon Bae, Jihoon Kim, Chanhun Choi, Changsu Na, Myeongkyu Park, Youngran Kim, Chang-Seob Seo, Seon-Jong Kim

**Affiliations:** 1 Yeongam Public Health Center, 39 Orijeong-gil, Yeongam, 58421 Jeollanam-do, Republic of Korea; 2 Department of Korean Medical Rehabilitation, Kwangju Korean Medicine Hospital of Dongshin University, 313 Baengnyeondae-ro, Mokpo, 58665 Jeollanam-do, Republic of Korea; 3 College of Korean Medicine, Dongshin University, 185 Geonjae-ro, Naju, 58245 Jeollanam-do, Republic of Korea; 4 Healthcare Research Institute, Korea Testing and Research Institute (KTR), 12-63 Sandan-gil, Hwasun, 58141 Jeollanam-do, Republic of Korea; 5 College of Pharmacy, Chonnam National University, 77 Yongbong-ro, Buk-gu, Gwangju, 61186 Republic of Korea; 6 Herbal Medicine Research Division, Korea Institute of Oriental Medicine, 1672 Yuseong-daero, Yuseong-gu, Daejeon, 34054 Republic of Korea

**Keywords:** ChondroT, 13-week repeated oral dose toxicity, 4-week recovery test, NOAEL

## Abstract

**Background:**

*ChondroT*, a new herbal medication, consists of *Angelica grosseserrata* Maxim., *Lonicera japonica* Thunb., *Angelica gigas* Nakai, *Clematis terniflora* var. *manshurica* (Rupr.) Ohwi, and *Phellodendron amurense* Rupr. (6:4:4:4:3). Our previous studies have shown that *ChondroT* exhibits significant anti-arthritic and anti-inflammatory effects. In this study, we aimed to assess the toxicological safety assessment of *ChondroT.*

**Methods:**

This study was designed to assess the safety of *ChondroT* after repeated oral administration.

Male and female Sprague-Dawley rats were treated with *ChondroT* at oral doses of 0, 500, 1000, and 2000 mg/kg for 13 weeks. Mortality, clinical signs, body weight changes, food consumption, ophthalmological findings, urinalysis, hematological and blood-chemical parameters, necropsy findings, organ weights, and histological markers were recorded throughout the study period. Rats were also monitored for an additional 4 weeks to determine the recovery time.

**Results:**

No death occurred and no significant changes in food consumption, ophthalmologic findings, and urinalysis were found. Although there were alterations in clinical signs, body weights, hematological parameters, blood-chemical parameters, necropsy findings, organ weights, and histological markers, they were not considered to be toxicologically significant.

**Conclusions:**

The results suggest that the no-observed adverse effects level (NOAEL) was 2000 mg/kg/day for the test substance. *ChondroT,* a new complex herbal medication composed of five plants, can therefore be used safely at the NOAEL.

## Background

*Ganghwaljetongyeum* (GHJTY) is a traditional Korean herbal medicine used to treat joint pain, restricted movement, fever, and swelling [1, 2]. In a previous study, bioinformatics and experimental screening were employed to improve the efficacy and relevance of GHJTY as a pharmaceutical [3], thus creating *ChondroT*, which consists of *Angelica grosseserrata* Maxim., *Lonicera japonica* Thunb., *Angelica gigas* Nakai, *Clematis terniflora* var. *mandshurica* (Rupr.) Ohwi, and *Phellodendron amurense* Rupr. (6:4:4:4:3).

*ChondroT* exerts chondroprotective effects and possesses multitarget mechanisms related to inflammation and arthritis [4–6]. In fact, the suppressive effect of *ChondroT* is greater than that of GHJT Y[7]. Moreover, *ChondroT* has shown therapeutic potential for the treatment of patients with hyperuricemia and gout [8]. Thus, it is currently undergoing clinical trials for approval from Ministry of Korean Food and Drug Safety [9].

Studies remain to be conducted on the toxicity of *ChondroT*. Although a 4-week repeated oral dose toxicity study had shown no adverse effects in Sprague-Dawley (SD) rats administered the maximum recommended dose of 2000 mg/kg/day via oral gavage [10], that study was performed primarily to determine the dosage range for a subsequent 13-week study. Thus, information regarding the oral toxicity of *ChondroT* during the subchronic period is insufficient.

In this 13-week repeated oral administration study on SD rats, we aimed to elucidate the toxic effects of *ChondroT*, identify its target organs, and determine its no-observed-adverse-effect-level (NOAEL) in order to provide toxicological data for assessing the safety of *ChondroT.*

## Methods

### Test facility

This study was conducted in compliance with the principles of Good Laboratory Practice at Korea Testing & Research Institute (KTR Hwasun) using the Korea Good Laboratory Practice (KGLP, Ministry of Food and Drug Safety Notification No. 2014-67, 2014-02-12) and OECD Principle of Good Laboratory Practice (ENV/MC/SHEM (98)17 as revised in 1997).

The study protocol was reviewed and approved (IAC2015-0797) by the Institutional Animal Care and Use Committee (IACUC) of KTR Hwasun based on the Animal Protection Act [Enforcement Date: 2015-01-20; No. 13023(2015-01-20, partial revision)] and the Laboratory Animal Act [Enforcement Date: 2013-07-30; No. 11987(2013-07-30, partial revision)].

The study was also conducted in accordance with the guideline for toxicity testing of pharmaceutical products by the Ministry of Food and Drug Safety (MFDS, Notification No. 2014-136, 2014-07-30).

### Animals

SD rats (Crl:CD, Specific Pathogen Free (SPF); age, 5 weeks old) were purchased from Orient Bio Co. Ltd. (Seongnam-si, Gyeonggi Province, Republic of Korea). A total of 110 rats (body weight: males, 121.4–141.6 g; females, 111.1–129.2 g) were quarantined and allowed to acclimatize for approximately one week.

After examining body weight changes and general conditions of animals, those deemed healthy were selected for this study (50 males, 50 females). Environmental conditions in the animal facility were maintained at 22 ± 3°C (20.5–23.27°C), 50 ± 20% (46.0–65.6%) relative humidity, 10–20 times/h ventilation frequency, 150–300 Lux luminous intensity, and 12-h light/12-h dark cycle. There were no influenceable variations with these conditions.

Animals were given irradiation-sterilized pellet food (Rodent Diet 20 5053, Labdiet, USA) and ultrafiltration (reverse osmotic, R/O) water via a water bottle, *ad libitum*. The absence of contamination was confirmed by a periodical analysis of the food manufacturing facility and KTR Hwasun for the R/O water.

After group assignment, the remaining animals (five males, five females) were sacrificed using carbon dioxide (CO_2_) in accordance with the American Veterinary Medical Association (AVMA) guidelines.

### Group assignment and dosing

After the acclimatization period, 100 healthy rats (6 weeks old; male 181.7–203.9 g, female 53.0–177.5 g) were randomly divided according to average weight and standard deviation. For dose selection, we considered the result of previously reported repeated toxicity study (Study No.: TBW-0064-15, 4 weeks repeated toxicity; 0, 500, 1000, 2000 mg/kg) [10] where no significant toxicological changes were found at doses up to 2000 mg/kg. The dose 2000 mg/kg was selected as the high (maximum) dose and two-fold intervals were used for the lower dose levels in the present 13-week repeated dose toxicity study; 1000 and 500 mg/kg were set as the intermediate and low (minimum) doses, respectively.

Study animals were divided into the groups listed in Table 1. Eighty rats were randomly divided into four groups (20 animals per group) for each dose (treatment group). Twenty rats were allocated to the control and high dose groups for the identification of any toxic effects, whether persistent or delayed, at 4 weeks post-exposure (recovery group).

**Table 1:** Dosage groups used for the 13-week repeated dose oral toxicity study with SD rats

Group	Dose (mg/kg/day)	Administered Fluid Volume (mL/kg)	Week	Number of rats(n)^*^ / Sex		
				Male	Female	Total
G1	0	10	13	10(1101-1110)	10(2101-2110)	20
			17^#^	5(1111-1115)	5(2111-2115)	10
G2	500	10	13	10(1201-1210)	10(2201-2210)	20
G3	1000	10	13	10(1301-1310)	10(2301-2310)	20
G4	2000	10	13	10(1401-1410)	10(2401-2410)	20
			17^#^	5(1411-1415)	5(2411-2415)	10

^*^ : Objective numbers, ^#^ : Recovery group

### Preparation of test substance and dosing

*ChondroT (*Lot No. 501) was supplied from Jung-woo Co. (Asan, Korea). The five herbs were combined in a 6:4:4:4:3 ratio (Table 2). *ChondroT* was prepared by carrying out water extraction once, using 10-fold solvent at 100°C for 3 h, and then filtering it (180 mesh). The water extract solution was concentrated using a continuous vacuum evaporator (approximately 55–60°C, 670 mmHg), and this was followed by vacuum drying using a vacuum drier (720 mmHg) for 8 h. The yield was approximately 29.4%.

**Table 2:** Composition of *ChondroT* and the used parts of five herbs

Latin name	Scientific name	Family	Used part	Rate	Source
Osterici Radix	*Ostericum koreanum* Maximowicz	Umbelliferae	Root	6	Korea
Angelicae Gigantis Radix	*Angelica gigas* Nakai	Umbelliferae	Root	4	Korea
Clematidis Radix	*Clematis manshurica* Ruprecht	Ranunculaceae	Root	4	China
Lonicerae Folium	*Lonicera japonica* Thunberg	Caprifoliaceae	Leaf	4	China
Phellodendri Cortex	*Phellodendron amurense* Ruprecht	Rutaceae	Tree bark	3	China

The *ChondroT* dried extract (light brown powder) was suspended in distilled water (D.W., Daihan Pharm Co., Ltd.) to achieve concentrations of 50, 100, and 200 mg/mL for treatment. The administration volume was 10 mL/kg based on the most recently measured body weight. The test substance was administered once/day, 7 days/week, for 13 weeks by oral gavage using a stomach tube and was suspended using a magnetic stirrer during administration. The control group was administered vehicle (DW, Daihan Pharm Co., Ltd.).

### High-Performance Liquid Chromatography (HPLC) analysis

Five marker components (chlorogenic acid, berberine chloride (Cl), oxypeucedanin hydrate, decursin, and decursinol angelate) were purchased from Acros Organics (Pittsburgh, PA, USA), Shanghai Sunny Biotech (Shanghai, China), ChemFaces (Wuhan, China), and NPC Bio Technology (Yeongi, Korea), respectively. Analytical method for quality control of *ChondroT* sample using HPLC was described in detail in previous studies [5, 6]. In other words, the HPLC used for simultaneous analysis of the five marker components was the Shimadzu Prominence LC-20A Series (Shimadzu, Kyoto, Japan) coupled with photo-diode array (PDA) detector. A SunFire^TM^ C_18_ analytical column (4.6 × 250 mm, 5 μm; Waters, Milford, MA, USA) was used for the separation of the five marker components at a constant temperature of 40°C. The mobile phase was eluted with distilled water (A) and acetonitrile (B), both containing 0.1% (v/v) formic acid under the gradient elution mode. The parameters for the HPLC quantitative analysis were as follows: 0–30 min, 10–100% B; 30–40 min, 100% B; 40–50 min, 100–10% B. The flow rate and injection volume were 1.0 mL/min and 10 μL, respectively.

### Observations

#### Clinical signs

All animals were observed daily before, during, and after oral treatment. Any treatment-related signs were individually recorded throughout the experimental period (13-week treatment and 4-week recovery period). In addition, mortality, development of clinical symptoms, and toxicity signs were recorded.

#### Body weight

Body weights of animals were measured on the day they were received, during group assignment, once per week, and the day before necropsy (13-week treatment and 4-week recovery period). Fasted weight was also measured on the day of necropsy to derive relative weight.

#### Food consumption

Food consumption was measured pre-dose and once per week for the 13-week treatment and 4-week recovery period. A weighed amount of food was placed in each cage and the amount remaining in each cage was measured the following day. Pre-dosing, food consumption was measured from group assignment to the first dosing day.

#### Ophthalmic examination and urinalysis

Ophthalmological examination and urinalysis were performed (five rats/sex/group) during the last week of treatment and the recovery period. Gross examination was performed followed by ophthalmological examination using mydriatic (Tropicamide 1%, Alcon, Belgium) and fundus camera (Genesis, Kowa, Japan). Urinalysis (i.e., appearance, volume, specific gravity, pH, protein, glucose, ketone body, bilirubin, urobilinogen, nitrite, blood, leukocyte, and urinary sediments) was performed with urine collected from a metabolic cage. Urinalysis items were analyzed using an automatic tester (CliniTek 500, Siemens, Germany), urine stick (Multistix 10 SG, Siemens, Germany) and a microscope (Leica, Germany).

#### Hematology and serum biochemistry

All animals were fasted overnight before blood sampling. Blood samples were drawn from the aorta under anesthesia and transferred to CBC bottle (EDTA K2, BD, USA) and vacutainer (9NC Sodium citrate, BD, USA) for hematological and coagulation tests, respectively. For the coagulation test, plasma was separated by centrifugation (3000 rpm, 4°C, 10 min). The hematological parameters, total erythrocyte count (red blood cells, RBC), hemoglobin concentration (HGB), hematocrit (HCT), mean corpuscular volume (MCV), mean corpuscular hemoglobin (MCH), mean corpuscular hemoglobin concentration (MCHC), reticulocyte (Retic), platelet count (PLT), total leucocyte count (white blood cells, WBC), WBC differential count (neutrophils, lymphocytes, monocytes, eosinophils, and basophils), prothrombin time (PT), and activated partial thromboplastin time (APTT) were measured using a hematological auto-analyzer (ADVIA 2120i, Siemens, USA) and coagulation analyzer (ACL 7000, Instrumentation Laboratory, USA).

The remaining samples, except blood samples for hematological and coagulation tests, were placed in tubes without an anticoagulant for serum separation. The tubes were kept at room temperature and serum was separated by centrifugation (3000 rpm, 4°C, 10 min). The biochemical parameters, total protein, albumin, albumin/globulin (A/G) ratio, total bilirubin (T-Bil; Hanlab Labmaster, Korea), alkaline phosphatase (ALP; Denka Seiken, Japan), aspartate aminotransferase (AST; Denka Seiken, Japan), alanine aminotransferase (ALT; Denka Seiken, Japan), creatinine (CREA; Hanlab Labmaster, Korea), blood urea nitrogen (BUN; Hanlab Labmaster, Korea), total cholesterol (T-CHO; Kyowa, Japan), triglyceride (TG; Kyowa, Japan), glucose (GLU; Denka Seiken, Japan), calcium (CA; Denka Seiken, Japan), inorganic phosphorus (IP; Hanlab Labmaster, Korea), creatine kinase (CK; Denka Seiken, Japan), sodium (Na^+^; Canon, Japan), potassium (K^+^; Canon, Japan), and chloride (Cl^-^; Canon, Japan) were analyzed using an autoanalyzer (TBA-120FR, Toshiba, Japan).

#### Gross finding at necropsy

On the day scheduled for necropsy, all animals were anaesthetized by isoflurane inhalation (Forane, JW Pharmaceutical, Korea) followed by blood sampling. Animals were exsanguinated from the posterior vena cava and aorta prior to necropsy. Macroscopic examinations of the external surface, all orifices, all internal organs of the head, thoracic and abdominal cavities were performed on the dead animals.

#### Organ weight

When all animals were sacrificed, absolute organ weights were measured and relative organ weights (organ weight to fasted body weight ratios before necropsy, %) were calculated for the following organs: brain, pituitary gland, heart, lung, liver, spleen, kidney (*), adrenal gland (*), testis (*), epididymis (*), ovary (*), uterus, thymus, prostate gland, and submandibular gland. The bilateral organs (*) were respectively measured and the measured weight was summed.

#### Histopathological examination

Tissues were fixed in neutral buffered 10% formalin, Bouin’s fixative (for the testes/epididymides) and Davidson’s solution (for the eyes with the Harderian gland). Tissues representing the liver, kidney, adrenal gland, heart, lung, brain (pituitary gland), spleen, prostate gland (with seminal vesicle and coagulating glands), testes, epididymides, ovary, uterus, vagina, tongue, trachea, esophagus, thymus, thyroid gland (with parathyroid gland), stomach, intestine, eye (with the Harderian gland), urinary bladder, submandibular gland, skin, pancreas, sternum, mammary gland, spinal cord, femur, mesenteric lymph node, sciatic nerve, and skeletal muscle from the control and high dose groups were embedded in paraffin, sectioned, stained with hematoxylin and eosin (H&E stain), and examined with a microscope. Necropsy was also performed with animals (No.1104, 1204, 1308, 2302).

#### Statistical Analysis

Data are presented as mean ± standard deviation (S.D.) for body weight, food consumption, organ weight, hematology, and biochemistry and were analyzed by SPSS software (ver. 19.0). The Leven’s test was performed to derive the homogeneity of variance and one way ANOVA was performed to determine the significant differences between groups. If there were no significant differences, an additional analysis was not performed; however, if a significant difference was confirmed, post-hoc test was used according to the result of variance homogeneity (homogeneity; Scheffe test, heterogeneity; Dunnett’s T3 test). In the recovery group, data analysis was performed using independent *t*-test. A *p* value < 0.05 was considered significant.

## Results

### Quality assessment of the five marker components in *ChondroT* Sample

**Fig. 1:**
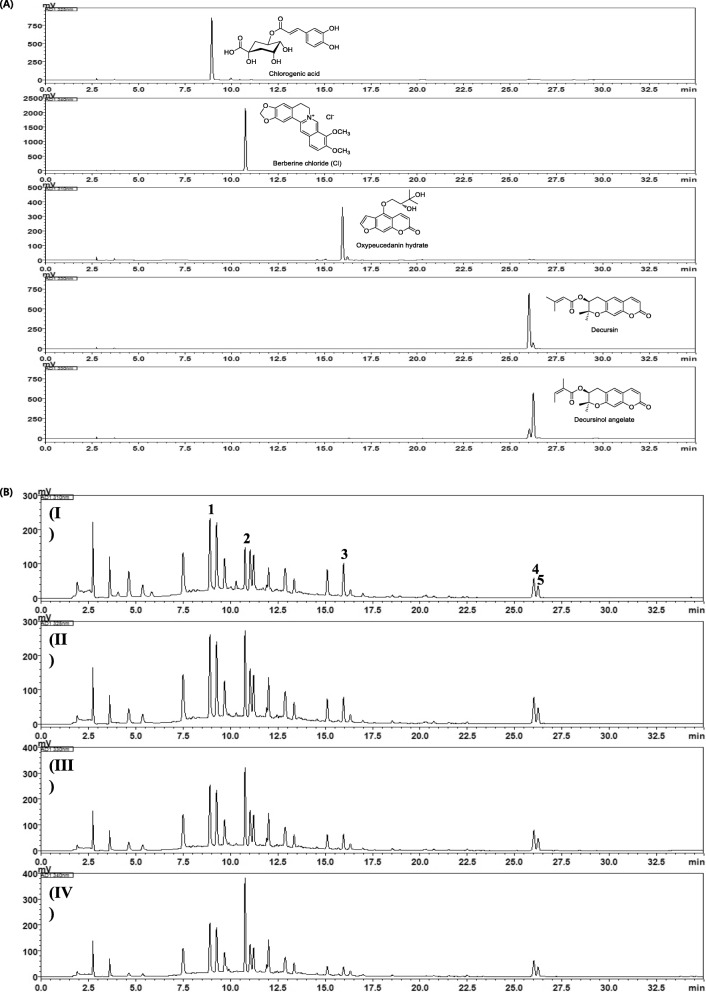
Typical HPLC-PDA chromatograms of each standard compound (**a**) and *ChondroT* sample at 310 (I), 325 (II), 330 (III), and 340 (IV) nm (**b**). Chlorogenic acid (1), Berberine Cl (2), oxypeucedanin hydrate (3), decursin (4), and decursinol angelate (5)

Using the established HPLC method, simultaneous determination was conducted to evaluate the 13-week treatment of *ChondroT* sample. The five marker components (chlorogenic acid, berberine Cl, oxypeucedanin hydrate, decursin, and decursinol angelate) were eluted within 30 min and the retention times of these components were 8.94, 10.80, 15.95, 26.02, and 26.24 min, respectively (Fig. 1). The coefficient of determination for the calibration curve of the five components was ≥ 0.9996, showing a good linearity. The amounts of the five marker components ranged between 0.81 and 5.46 mg/g (Table 3).

**Table 3:** Parameters for quantitative analysis and amount of five marker components in *ChondroT* sample

Compound	Detection wavelength (nm)	Linear range (μg/mL)	Regression equation^a^	*r*^2^	Amount (mg/g)
Chlorogenic acid	325	1.56−100.00	*y* = 30429.94*x* – 42881.66	0.9996	5.46
Berberine Cl	340	0.78−50.00	*y* = 81886.13*x* + 16338.31	0.9999	1.87
Oxypeucedanin hydrate	310	1.56−100.00	*y* = 26221.17*x* + 5076.06	1.0000	2.03
Decursin	330	0.78−50.00	*y* = 37915.21*x* + 52952.92	1.0000	1.09
Decursinol angelate	330	0.78−50.00	*y* = 28967.64*x* + 29015.53	1.0000	0.81

### Mortality and clinical signs

During the experimental period, treatment-related moribund, dead animals, and clinical signs were not observed for both the sexes in the control and treatment groups. However, loss of fur in posterior neck from day 12 to 91 and abdomen from day 25 to 91 (one female at middle dose) and a nodule (slightly hard with an egg-shape) from day 63 to 91 (one female administered the middle dose) were clinical signs observed. Salivation was transiently or intermittently observed in both the sexes when the high dose was administered (Table 4).

**Table 4:** Clinical signs observed in the 13-week repeated oral dose toxicity study with SD rats

Group (Dose)^*^	Week	Sex	Number of rats	Weeks / Number of rats observed clinical signs																	Mortality (dead/total)
				1	2	3	4	5	6	7	8	9	10	11	12	13	14	15	16	17	
G1 (0)	13	Male	10	0	0	0	0	0	0	0	0	0	0	0	0	0	-	-	-	-	0%
		Female	10	0	0	0	0	0	0	0	0	0	0	0	0	0	-	-	-	-	0%
	17^#^	Male	5	0	0	0	0	0	0	0	0	0	0	0	0	0	0	0	0	0	0%
		Female	5	0	0	0	0	0	0	0	0	0	0	0	0	0	0	0	0	0	0%
G2 (500)	13	Male	10	0	0	0	0	0	0	0	0	0	0	0	0	0	-	-	-	-	0%
		Female	10	0	1^**^	1^**^	1^**^	1^**^	1^**^	1^##^	1^##^	2^##^	2^##^	2^##^	2^##^	2^##^	-	-	-	-	0%
G3 (1000)	13	Male	10	0	0	0	0	0	0	0	0	0	0	0	0	0	-	-	-	-	0%
		Female	10	0	0	0	0	0	0	0	0	0	0	0	0	0	-	-	-	-	0%
G4 (2000)	13	Male	10	0	0	0	0	0	0	0	0	0	0	0	0	0	-	-	-	-	0%
		Female	10	0	0	0	0	0	0	0	0	0	0	0	0	0	-	-	-	-	0%
	17^#^	Male	5	0	0	0	0	0	0	0	0	0	0	0	0	0	0	0	0	0	0%
		Female	5	0	0	0	0	0	0	0	0	0	0	0	0	0	0	0	0	0	0%

^*^ : mg/kg, - : not applicable, ^#^ : Recovery group, ^**^ : Loss of fur of posterior neck and abdomen(No.2308), ^##^ : Loss of fur of posterior neck and abdomen(No.2308), Slightly hard egg-shaped nodule of right neck(No. 2302)

### Body weight and food consumption

**Fig. 2:**
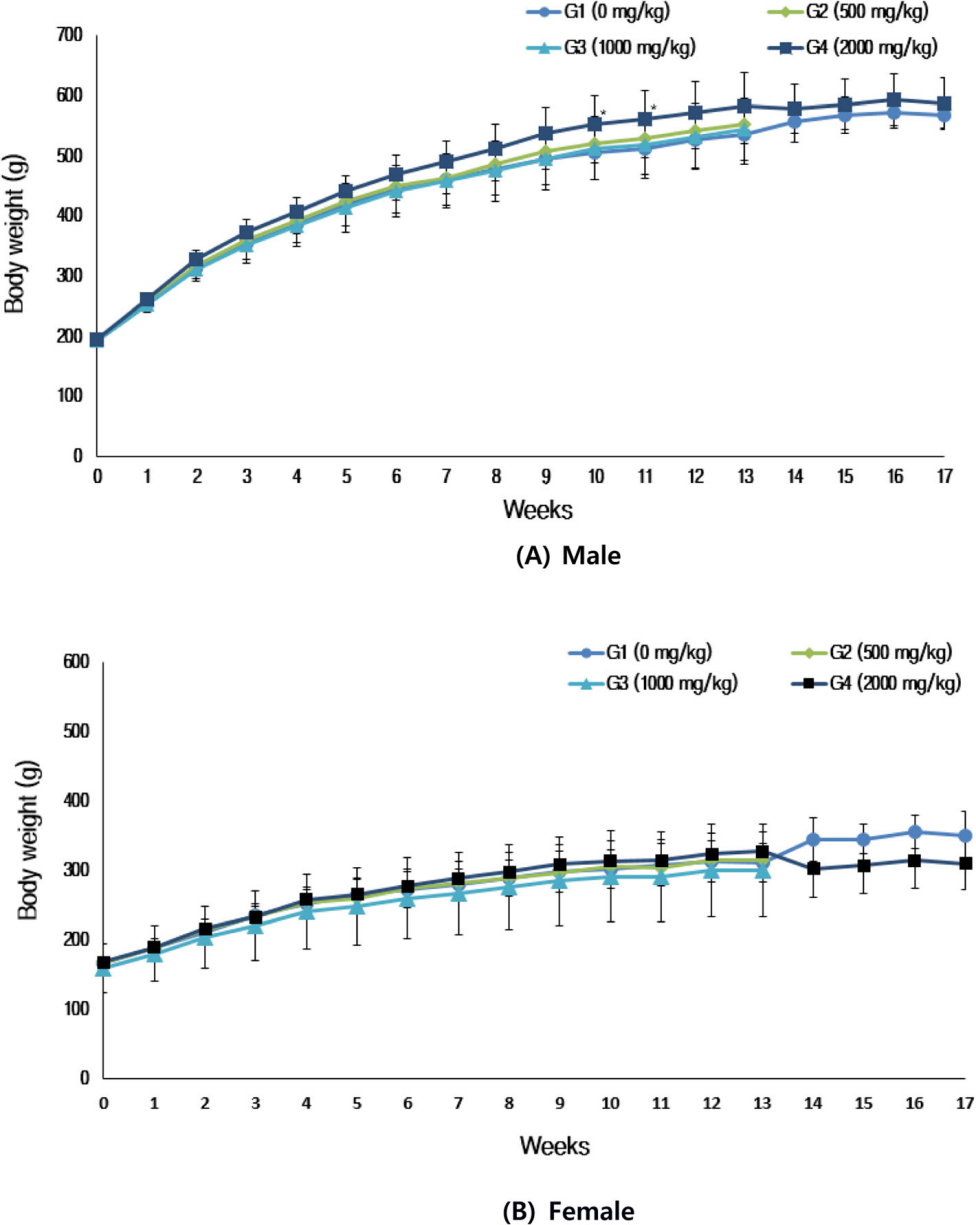
Body weights of the rats used in the experiment

A gradual increase in body weight of males was observed over the treatment period, with significant differences (*p* < 0.05) at weeks 10 (ANOVA test: F value, 3.345; p value, 0.027; post-hoc test: Scheffe p value, 0.041) and 11 (ANOVA test: F value, 3.615; p value, 0.020; post-hoc test: Scheffe *p* value, 0.036) for animals administered the 2000 mg/kg dose. No significant differences were found for females among the groups (Fig. 2).

**Fig. 3:**
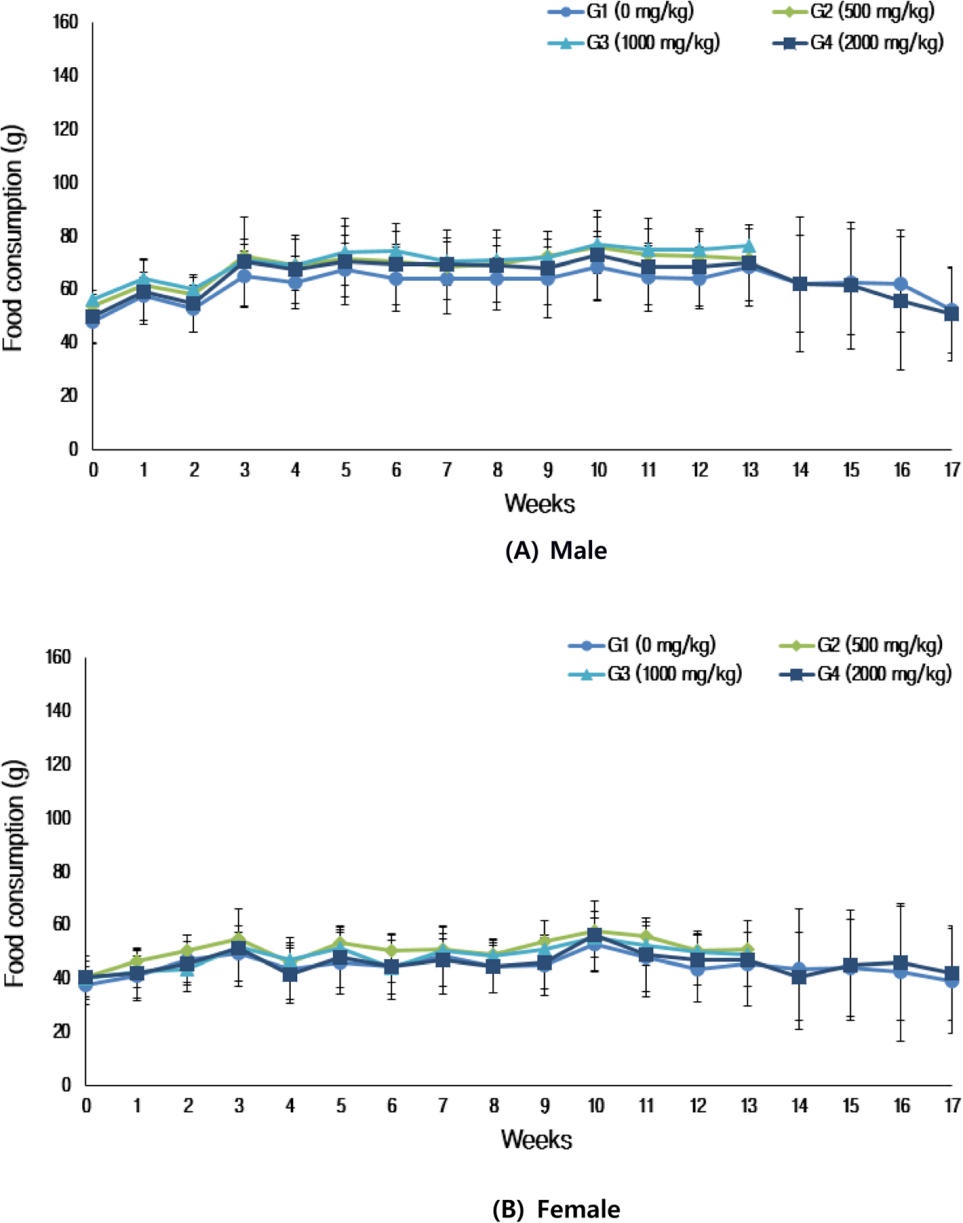
Food consumption of the rats used in the experiment

There was no significant difference in food consumption between the treatment groups and control group for both the sexes (Fig. 3).

### Ophthalmological examination and urinalysis

There were no treatment-related changes during the treatment and recovery periods for the different dosage groups.

### Hematology and serum biochemistry

There was no significant difference in the hematological items over the treatment period. However, some items displayed significant differences in the recovery period. There was a significant increase in mono (Levene’s test: F value, 1.884; *p* value, 0.212; independent *t*-test: *p* value, 0.049) for males and neut (Levene’s test: F value, 0.007; *p* value, 0.937, independent *t*-test: *p* value, 0.036) and baso (Levene’s test: F value, 0.099, *p* value: 0.762; independent *t*-test: *p* value, 0.020) for females administered the 2000 mg/kg dose, and a significant decrease in Lymph (Levene’s test: F value, 0.821; *p* value: 0.395, independent *t*-test: *p* value, 0.038) for females administered the 2000 mg/kg dose. Samples with a blood clot were excluded from the statistical analysis (treatment group, one male administered 2000 mg/kg; recovery group, one male and one female in the control group; Table 5).

**Table 5:** Hematological parameters

Parameters	Group(Dose)^*^	G1(0)		G2(500)	G3(1000)	G4(2000)	
	Sex/Week	13week	17week^#,**^	13week	13week^##^	13week^**^	17week^#^
Total leucocyte count (10^3^cells/μL)	Male	5.09±1.38	6.55±2.37	5.39±1.13	6.15±2.13	6.27±3.02	5.86±1.39
	Female	3.18±0.56	3.00±1.07	2.66±0.80	5.10±5.41	4.16±0.66	2.89±1.08
Total erythrocyte count (10^6^cells/μL)	Male	8.44±0.33	7.97±0.48	8.38±0.21	8.16±0.79	8.16±0.94	8.17±0.35
	Female	7.78±0.35	7.24±0.10	7.53±0.34	7.55±0.45	7.45±0.24	7.30±0.15
Hemoglobin concentration (g/dL)	Male	14.6±0.7	14.0±0.7	14.4±0.4	14.5±1.2	14.2±1.4	14.3±0.4
	Female	14.2±0.6	13.5±0.3	13.8±0.6	13.8±0.8	13.7±0.4	13.8±0.3
Hematocrit(%)	Male	42.2±1.7	40.1±2.0	42.1±1.0	42.0±4.1	41.0±4.1	41.0±1.1
	Female	41.1±1.7	38.6±0.5	40.0±1.6	40.1±1.9	39.5±0.9	39.4±0.9
Mean corpuscular volume (fL)	Male	50.0±1.5	50.3±0.9	50.3±1.6	51.5±1.3	50.4±1.4	50.3±1.5
	Female	52.9±1.4	53.3±0.5	53.2±1.5	53.1±1.4	53.2±2.0	54.1±1.0
Mean corpuscular hemoglobin (pg)	Male	17.2±0.6	17.6±0.3	17.2±0.6	17.8±0.5	17.4±0.6	17.5±0.6
	Female	18.3±0.4	18.7±0.4	18.4±0.7	18.2±0.5	18.4±0.7	18.9±0.1
Mean corpuscular hemoglobin concentration(g/dL)	Male	34.5±0.5	35.0±0.5	34.2±0.4	34.5±0.6	34.6±0.5	34.9±0.4
	Female	34.6±0.3	35.1±0.7	34.6±0.6	34.4±0.4	34.6±0.4	35.0±0.5
Reticulocyte(10^9^cells/μL)	Male	155.3±45.7	169.5±60.4	164.2±34.1	160.0±32.8	200.1±95.6	184.8±19.0
	Female	150.4±40.2	172.6±34.6	142.1±29.5	176.4±72.4	179.2±31.4	146.6±26.4
Reticulocyte(%)	Male	1.84±0.53	2.14±0.83	1.96±0.32	1.95±0.32	2.43±1.03	2.26±0.21
	Female	1.94±0.51	2.39±0.51	1.89±0.37	2.37±1.10	2.41±0.45	2.01±0.34
Platelet(10^3^cells/μL)	Male	1041.3±142.1	830.0±431.1	973.9±73.6	849.6±306.5	819.6±452.8	1035.6±70.1
	Female	967.6±68.5	741.6±402.8	844.3±300.9	1008.5±99.2	969.0±85.8	1006.8±99.1
Prothrombin time(sec)	Male	13.6±0.8	13.1±0.4	14.5±1.1	14.9±1.6	14.0±1.9	12.7±0.4
	Female	12.6±0.4	13.4±1.9	12.4±0.3	12.4±0.4	12.3±0.3	12.9±0.2
Activated partial thromboplastin time(sec)	Male	17.7±1.7	17.7±2.6	20.1±1.7	19.4±1.9	19.0±3.2	15.9±2.8
	Female	14.6±1.4	14.8±3.6	14.8±1.2	14.4±1.5	14.1±1.6	16.5±2.0
Neutrophils(%)	Male	21.2±10.0	16.2±6.9	20.4±6.4	18.9±6.4	17.9±4.4	17.8±3.2
	Female	19.9±7.6	16.4±2.8	15.7±4.8	26.4±16.7	15.3±4.3	22.3±3.1^†^
Lymphocytes(%)	Male	73.1±11.3	78.2±7.8	73.1±7.3	76.3±7.5	76.9±5.0	75.7±4.2
	Female	73.0±7.7	76.5±3.3	78.3±5.3	67.1±15.9	79.2±5.1	68.5±4.9^†^
Monocytes(%)	Male	2.2±1.0	1.6±0.2	2.4±0.5	1.8±0.7	2.2±1.1	2.1±0.4^†^
	Female	2.4±0.3	2.1±0.7	1.7±0.8	2.1±0.5	1.8±0.5	1.9±0.5
Eosinophils(%)	Male	1.9±0.8	2.8±1.2	2.0±1.0	1.5±0.7	1.6±0.4	3.5±0.7
	Female	3.0±1.0	4.0±1.7	3.0±1.6	3.3±1.6	2.3±0.6	6.6±3.1
Basophils(%)	Male	0.1±0.1	0.2±0.1	0.2±0.1	0.2±0.1	0.2±0.0	0.1±0.1
	Female	0.1±0.1	0.1±0.3	0.1±0.1	0.1±0.1	0.1±0.1	0.1±0.0^†^

Values are in Mean±Standard deviation, ^*^ : mg/kg, ^#^ : Recovery group, ^**^ : blood coagulation(No. 1111, 1409, 2111. except for Prothrombin time and Activated partial thromboplastin time), ^##^ : There was nodule with slightly hard egg-shaped(No. 2302), ^†^ : significant difference as compared with control(*p*<0.05)

For serum biochemistry items, significant increases were found for sodium (ANOVA test: F value, 4.475; *p* value, 0.009; post-hoc test: Scheffe *p* value, 0.026) in males and triglycerides (ANOVA test: F value, 3.443, *p* value, 0.027, post-hoc test, Scheffe *p* value, 0.047) in females administered 2000 mg/kg. In the recovery group, no significant changes were found for both the sexes when administered 2000 mg/kg (Table 6).

**Table 6:** Blood chemical parameters

Parameters	Group(Dose)^*^	G1(0)		G2(500)	G3(1000)	G4(2000)	
	Sex/Week	13week	17week^#^	13week	13week	13week	17week^#^
Total protein(g/dL)	Male	5.9±0.4	5.8±0.3	5.9±0.3	6.1±0.3	6.1±0.1	6.0±0.3
	Female	6.4±0.5	6.6±0.5	6.7±0.5	6.5±0.5	6.6±0.4	6.4±0.3
Albumin(g/dL)	Male	3.8±0.2	3.9±0.2	3.8±0.1	3.9±0.2	4.0±0.1	3.9±0.2
	Female	4.2±0.3	4.4±0.3	4.4±0.3	4.3±0.3	4.4±0.2	4.2±0.3
A/G ratio	Male	1.9±0.1	2.0±0.1	1.9±0.1	1.8±0.1	1.9±0.1	1.8±0.1
	Female	2.1±0.1	2.1±0.2	1.9±0.1	2.0±0.1	2.0±0.1	1.9±0.1
Total bilirubin(mg/dL)	Male	0.0±0.0	0.04±0.01	0.0±0.0	0.0±0.0	0.0±0.0	0.03±0.01
	Female	0.0±0.0	0.05±0.04	0.1±0.0	0.1±0.1	0.1±0.0	0.05±0.02
Alkaline phosphatase(U/L)	Male	305.3±66.1	240.4±55.7	279.8±50.9	294.6±48.2	259.4±50.4	236.8±17.4
	Female	145.8±46.6	108.0±51.9	166.2±73.0	168.9±70.8	150.6±44.0	135.8±40.1
Aspartate aminotransferase(U/L)	Male	97.0±21.9	130.2±42.4	102.1±23.9	107.0±35.7	105.9±36.9	85.4±8.4
	Female	91.8±22.4	123.8±23.6	105.0±24.2	87.9±21.2	87.4±23.7	113.8±9.1
Alanine aminotransferase(U/L)	Male	35.1±7.9	46.0±15.9	32.5±5.9	31.0±5.5	32.2±5.8	30.8±6.6
	Female	35.8±10.8	36.4±7.8	37.7±10.7	33.0±12.0	31.4±9.8	48.8±14.6
Creatinine(mg/dL)	Male	0.4±0.0	0.5±0.0	0.4±0.0	0.4±0.0	0.4±0.0	0.4±0.1
	Female	0.5±0.1	0.5±0.0	0.5±0.0	0.5±0.1	0.5±0.1	0.5±0.0
Blood urea nitrogen(mg/dL)	Male	15.0±1.4	14.1±1.4	13.0±1.8	14.1±2.7	14.6±2.1	14.5±1.2
	Female	16.4±3.0	19.7±4.7	16.9±3.6	16.4±3.0	16.9±2.0	16.5±1.2
Total cholesterol(mg/dL)	Male	56.7±19.7	58.6±14.4	58.4±11.8	56.7±17.7	65.0±15.2	77.6±19.2
	Female	59.0±15.3	63.6±10.4	69.7±12.3	76.4±28.2	70.7±11.2	73.4±6.9
Triglycerides(mg/dL)	Male	22.0±12.1	30.2±22.3	26.3±5.8	25.2±8.0	32.8±14.3	37.0±11.6
	Female	14.2±4.9	26.2±14.3	23.1±10.9	18.6±7.7	25.4±9.2^**^	18.0±3.3
Glucose(mg/dL)	Male	153.5±18.6	174.4±18.2	160.6±25.1	151.1±17.6	153.0±21.2	191.4±37.0
	Female	144.6±14.4	162.8±17.6	158.4±11.7	168.0±32.6	156.3±12.8	162.0±18.7
Calcium(mg/dL)	Male	9.3±0.4	8.8±0.1	9.3±0.3	9.3±0.3	9.6±0.2	8.9±0.1
	Female	9.3±0.4	9.0±0.3	9.5±0.3	9.4±0.4	9.4±0.3	8.9±0.2
Inorganic phosphorus(mg/dL)	Male	6.1±0.6	5.1±0.3	5.9±0.4	5.7±0.6	5.9±0.4	5.0±0.4
	Female	4.5±1.0	3.7±0.9	4.9±1.1	4.7±1.0	4.7±1.0	3.3±0.5
Creatine kinase(U/L)	Male	305.8±184.8	392.8±184.1	402.7±266.3	461.3±412.7	414.2±408.6	262.0±112.5
	Female	206.5±135.2	525.8±260.9	292.1±267.7	192.0±80.9	222.7±139.0	384.8±260.2
Sodium(mmol/L)	Male	145.7±0.9	146.3±1.0	145.7±0.6	146.1±0.8	146.7±0.6^**^	145.9±1.4
	Female	144.4±1.3	145.3±0.8	144.8±0.9	144.9±0.0	143.8±1.0	144.8±0.4
Potassium(mmol/L)	Male	4.7±0.2	4.5±0.3	4.7±0.3	4.5±0.4	4.6±0.4	4.5±0.5
	Female	3.9±0.2	4.31±0.35	4.2±0.6	4.2±0.3	4.1±0.2	4.52±0.23
Chloride(mmol/L)	Male	106.9±1.7	106.6±1.0	105.8±0.9	106.4±1.0	106.0±1.4	106.4±2.1
	Female	107.2±1.4	107.8±1.0	106.8±2.1	106.2±1.6	106.0±1.9	108.1±1.1

Values are in Mean±Standard deviation, ^*^ : mg/kg, ^#^ : Recovery group, ^**^ : significant difference as compared with control(*p*<0.05)

### Gross finding at necropsy

At the end of treatment, there were no treatment-related gross findings in the treatment groups over the 13-week treatment period and 4-week recovery period. However, the following gross findings were observed in the treatment group: reduced size of the left testis in one male in the control group, yellowish nodule on the prostate gland of one male in the low dose group and cysts and mass (3.0 × 4.5 × 3.0 cm) in the right submandibular region in one female administered the middle dose.

### Organ weight

For absolute organ weight in the treatment group, there was a significant increase in the weight of the heart (low dose, ANOVA test: F value, 6.205; p value, 0.002; post-hoc test: Scheffe *p* value, 0.038), liver (high dose, ANOVA test: F value, 4.273; *p* value, 0.011; post-hoc test: Scheffe *p* value, 0.016) and lung (high dose, ANOVA test: F value, 4.559; *p* value, 0.008; post-hoc test, Scheffe *p* value, 0.021) of males; these changes did not resemble that of relative organ weight. For relative organ weight, a significant decrease was found for the brain (high dose, ANOVA test: F value, 4.248; *p* value, 0.11; post-hoc test : Scheffe *p* value, 0.018) of a male. There were no significant changes in the organ weight of females in the different treatment groups.

In the recovery group, there was a significant increase in the absolute organ weight of the thymus (high dose, Levene’s test: F value, 0.469; *p* value, 0.513; independent *t*-test, *p* value, 0.013) of males and a significant decrease for that of the kidney (bilateral weight, high dose, Levene’s test: F value, 4.007; *p* value, 0.080; independent *t*-test: *p* value, 0.029) and brain (high dose, Levene’s test: F value, 0.063; *p* value, 0.808; independent *t*-test: *p* value, 0.021) of females. Similarly, a significant decrease in relative organ weight was found for the testis (bilateral weight, high dose, Levene’s test: F value, 1.281; *p* value, 0.291; independent *t*-test: *p* value, 0.040).

### Histopathological examination

The following histopathological lesions were observed in the high dose treatment group: inflammatory cell foci in the liver (one male), tubular basophilia (two males, one female), medullary mineralization (two females) in the kidney, cortical vacuolation of the adrenal gland (four males), inflammatory cell infiltration of the prostate gland (interstitial, one male), and cysts in the ovary (one female). Some of these lesions were also observed in the control group (i.e., inflammatory cell foci in the liver, tubular basophilia and medullary mineralization in the kidney, cortical vacuolation in the adrenal gland, and inflammatory cell infiltration/interstitial in the prostate gland).

The following histopathological lesions were observed in the high dose recovery group: medullary mineralization in the kidney (one female), cysts in the pituitary gland (pars distalis, one male), and inflammatory cell infiltration in the prostate gland (interstitial, one male). Similar to the treatment groups, some of these lesions were observed in the control group (i.e., medullary mineralization in the kidney and cysts in the pars distalis of the pituitary gland).

**Fig. 4:**
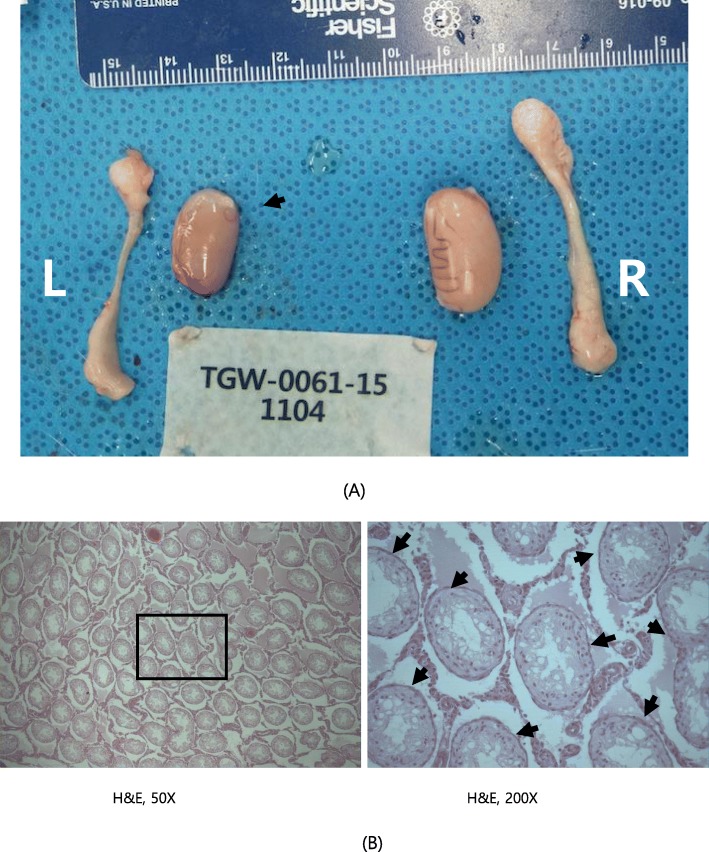
Reduced left testis in male G1 (control group; rat number 1104) (**a**) Necropsy finding, (**b**) Tubular atrophy; H&E 50X, 200X

**Fig. 5:**
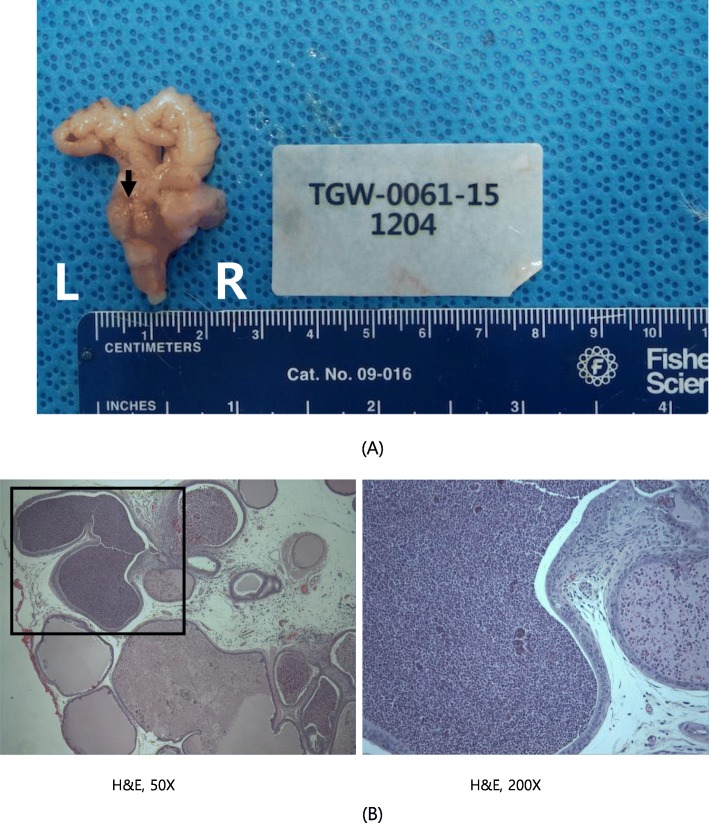
Yellowish nodules on the prostate gland of male G2 (low dose group; rat number 1204) (**a**) Necropsy finding, (**b**) Abscess; H&E 50X, 200X

**Fig. 6:**
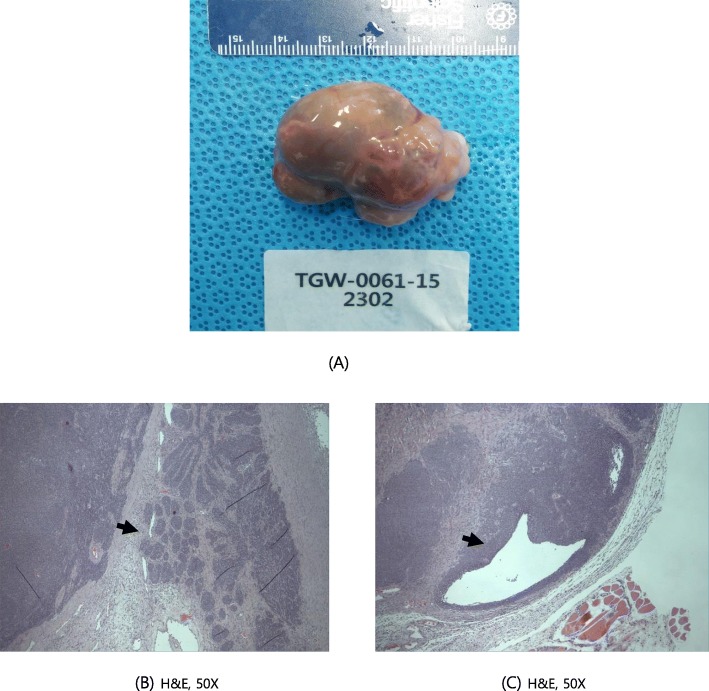
Cysts and mass in the submandibular region of female G3 (middle dose group; rat number 2302) (**a**) Necropsy finding (**b**) Basal cell carcinoma with aggregates of basaloid cells, H&E 50X (C) Basal cell carcinoma with cystic structure, H&E 50X

**Fig. 7:**
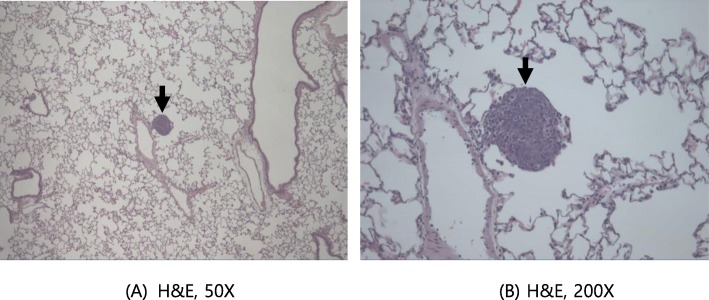
Basal cell carcinoma metastasis in the lung (tumor cell nodule) of female G3 (middle dose group; rat number 2302) (**a**) H&E 50X (**b**) H&E 200X

Moreover, histopathological lesions were also observed in organs with gross lesions. These include tubular atrophy in the testis, oligospermia in the epididymis (reduced left testis in the control group, Fig. 4), abscess (yellowish nodule on the prostate gland of animals administered the middle dose, Fig. 5), and basal cell carcinoma (cysts and mass in the right submandibular region of animals administered the middle dose, Fig. 6). Lung metastasis was also observed following basal cell carcinoma (Fig. 7). The histopathological lesions are shown in Table 7.

**Table 7:** Histopathological findings

Organs	Histopathological findings	G1(0)^*^				G4(2000)^*^			
		13week		17week^#^		13week		17week^#^	
		M	F	M	F	M	F	M	F
Liver	NAD^**^	8	9	4	5	9	10	5	5
	Inflammatory cell foci - Minimal, multifocal	2	1	1	0	1	0	0	0
Kidney	NAD	9	8	4	2	8	7	5	4
	Tubular basophilia - Minimal, multifocal	1	2	1	0	1	1	0	0
	Tubular casts - present	1	0	0	0	0	0	0	0
	Medullary mineralization - Minimal, multifocal	0	1	0	2	0	2	0	1
	Focal nephropathy - Slight, focal	0	0	1	0	0	0	0	0
	Inflammatory cell infiltration, interstitial - Minimal, Multifocal	0	0	0	1	0	0	0	0
Adrenal Gland	NAD	7	10	5	5	6	10	5	5
	Cortical vacuolation - Minimal, multifocal	3	0	0	0	4	0	0	0
Heart	NAD	10	10	5	5	10	10	5	5
Lung (bronchus)	NAD	10	10	4	5	10	10	5	5
	Inflammatory cell infiltration, perivascular - Minimal, multifocal	0	0	1	0	0	0	0	0
Brain^##^	NAD	10	10	4	5	10	10	4	5
	Pituitary: Cyst, pars distalis - Present	0	0	1	0	0	0	1	0
Spinal Cord	NAD	10	10	5	5	10	10	5	5
Spleen	NAD	10	10	5	5	10	10	5	5
Seminal Vesicle	NAD	10	-	5	-	10	-	5	-
Prostate gland	NAD	9	-	5	-	9	-	4	-
	Inflammatory cell infiltration, interstitial - Minimal, Multifocal	1	-	0	-	1	-	1	-
Testis	NAD	9	-	5	-	10	-	5	-
	Tubular atrophy in left testis - Severe, diffuse	1	-	0	-	0	-	0	-
Epididymis	NAD	9	-	5	-	10	-	5	-
	Oligospermia in left epididymis - Severe, diffuse	1	-	0	-	0	-	0	-
Ovary	NAD	-	9	-	4	-	9	-	5
	Cyst - Present	-	1	-	1	-	0	-	0
	Cysts - Present	-	0	-	0	-	1	-	0
Uterus	NAD	-	10	-	5	-	10	-	5
Vagina	NAD	-	10	-	5	-	10	-	5
Trachea	NAD	10	10	5	5	10	10	5	5
Esophagus	NAD	10	10	5	5	10	10	5	5
Thymus	NAD	10	10	5	4	10	10	5	5
	Cysts - Present	0	0	0	1	0	0	0	0
Thyroid gland	NAD	10	10	5	5	10	10	5	5
Tongue	NAD	10	10	5	5	10	10	5	5
Stomach	NAD	10	10	5	5	10	10	5	5
Urinary bladder	NAD	10	10	5	5	10	10	5	5
Intestine^†^	NAD	10	10	5	5	10	10	5	5
Eye	NAD	10	10	5	5	10	10	5	5
Harderian gland	NAD	10	10	5	5	10	10	5	5
Skin	NAD	10	10	5	5	10	10	5	5
Mammary gland	NAD	10	10	5	5	10	10	5	5
Submandibular gland	NAD	10	10	5	5	10	10	5	5
Skeletal muscle	NAD	10	10	5	5	10	10	5	5
Sciatic nerve	NAD	10	10	5	5	10	10	5	5
Pancreas	NAD	10	10	4	5	10	10	5	5
	Islet fibrosis - Minimal, multifocal	0	0	1	0	0	0	0	0
Sternum	NAD	10	10	5	5	10	10	5	5
Femur	NAD	10	10	5	5	10	10	5	5

^*^ : Dose(mg/kg), ^#^ : Recovery group ^**^ : No abnormality detected, ^##^ : Cerebrum, Cerebellum, Pituitary, ^†^ : Both small and large intestine

## Discussion

Osteoarthritis is emerging as an important issue resulting from the loss of cartilage associated with the shape of bones, such as osteophytes and subchondral bone sclerosis. It is caused by population aging, an increase in obesity, and a lack of definitive treatment [11–13]. Symptoms of osteoarthritis include progressive asymmetrical pain, joint stiffness, and restricted joint movement, which can hinder the performance of daily activities [14–16].

*ChondroT* is a new complex herbal medication developed for the treatment of osteoarthritis and its efficacy and safety have been demonstrated in several studies [4–8]. Although the safety of this herbal complex has been confirmed through animal studies, we sought to carry out the present study with additional doses and perform a rigorous toxicity study that could be translated to human clinical trials.

Herein, we aimed to evaluate the systemic toxicity response when *ChondroT*, the test substance, is administered via the oral route for 13 weeks, and determine its NOAEL. This 13-week repeated oral administration study included doses of 500, 1000, and 2000 mg/kg body weight.

During the test period, there were no treatment-related moribund or dead animals of any sex in the treatment groups. In addition, there were no significant changes in food consumption, urinalysis, and ophthalmological examination. Clinical signs included loss of fur in one female administered the 1000 mg/kg dose; however, this was not dose-dependent and was only observed in one animal. Although salivation was observed in some of the animals administered 2000 mg/kg of the test substance, continuous and transient or intermittent observations were not found for both sexes. Hence, these signs did not represent treatment-related toxicological effects and were considered to be incidental signs.

Body weight is a parameter affected by adverse effects exhibited by drugs and chemicals [17]. The increasing tendency of body weight in males administered the 2000 mg/kg dose was considered to be a treatment-related effect and not a toxicological effect. This was due to the changes observed in organ weight, hematological biochemistry parameters, and histopathological examination. A further study may however be needed to confirm whether or not toxic effects have an impact on body weight (e.g., chronic study).

There were no significant differences among the treatment groups based on the hematological analysis. Although significant differences were found when the 2000 mg/kg dose was administered, no histological lesions, organ weight changes, and gross findings were observed in the relevant organs. This indicates a normal biological variation and the lack of a treatment-related effect.

Based on the necropsy findings, there were no treatment-related effects for both sexes in the treatment groups. Nonetheless, histopathological examination revealed gross findings such as tubular atrophy with oligospermia of the epididymis and abscess in the control (reduced left testis) and treatment groups (nodule on the prostate gland at 500 mg/kg), respectively.

Previously, the incidence of spontaneous testicular atrophy in the testis of SD rats was reported to be 0.2%, 7.9%, and 10% at 4, 13, and 26 weeks, respectively. In addition, the epididymis with testicular atrophy has been reported to show low sperm density (oligospermia) [18]. Therefore, the observed lesion in this study may have been an aberration based on the lesion observed in the control group.

Male accessory sex glands are frequently observed as a site of inflammatory lesions and may appear as large abscesses or small suppurative lesions. Prostatitis is commonly observed in older rats but has been observed in young adult rats used in toxicity studies, especially in the dorsolateral prostate gland [19]. Therefore, the gross finding for the prostate gland in animals in the middle dose group might not be a treatment-related effect due to the non-existent dose relationship and the frequency of necropsy.

Organ weight could be a meaningful indicator of treatment-related changes with or without corresponding histopathological examination in repeated toxicity studies [20]. In this study, organ weight changes lacking a dose relationship were observed in some organs. In addition, a relationship between absolute weight and relative weight was not found. Additionally, treatment-related histological findings were not observed. Therefore, changes were considered to be normal biological variation.

In the histopathological examination, the lesions observed in animals administered the 2000 mg/kg were spontaneous incidence lesions, such as inflammatory cell foci (liver), tubular basophilia/medullary mineralization (kidney), cortical vacuolation (adrenal gland), inflammatory cell infiltration (prostate gland), and cysts (ovary, pars distalis of pituitary gland), which are common in repeated toxicity studies. These lesions were also observed in the control group but their frequency and grade were minor [19]. In addition, these lesions might have been background lesions and not a treatment-related effect.

Spontaneous basal cell carcinoma is very rare in rats, with an incidence rate of only 0.14% in aged animals. It is also rarely observed in young SD rats. Spontaneous basal cell carcinoma was reported recently in a subcutaneous mass found in the left inguinal region of a young (7-week) SD rat [21, 22]. In the present study, this lesion was only observed in one animal in the middle dose group and did not result in a dose-dependent response. Therefore, it was considered to be a treatment-related effect and spontaneous lesion.

Sub-chronic exposure of rats to *ChondroT* did not result in treatment-related changes at doses up to 2000 mg/kg. This result suggests that *ChondroT* might be safe and is in agreement with the human equivalent dose (HED) of 324.32 mg/kg/day; animal dose (mg/kg) x (rat K_m_ factor ‘6’/Human adult 60 kg K_m_ factor ‘37’)] using body surface area (BSA, mg/m^2^) for initial clinical trials involving healthy adult volunteers [23, 24].

In conclusion, the results of the present 13-week repeated oral dose toxicity study demonstrated that administering *ChondroT* did not adversely affect most of the toxicological factors in male and female SD rats. With the experimental conditions adopted in the present study, we found that the NOAEL of *ChondroT* was 2000 mg/kg/day and a target organ was not indicated.

## Conclusions

In previous studies, *ChondroT* was demonstrated to be effective in arthritis via its chondroprotective and anti-inflammatory mechanisms. Based on the results of this study, we found that its NOAEL was 2000 mg/kg, which reveals that *ChondroT* does not exhibit toxicity under the conditions employed in the study. As a new complex herbal medication composed of five plants, *ChondroT* can be used safely at its NOAEL

## Data Availability

The datasets used and/or analyzed in the current study are available from the corresponding author on reasonable request.

## Abbreviations

GHJTY: Ganghwaljetongyeum
OA: Osteoarthritis
CFA: Complete Freund’s adjuvant
TNF-α: Tumor necrosis factor-α
IL-1β: Interleukin-1β
AST: Aspartate aminotransferase
ALT: Alanine transaminase
BUN: Blood urea nitrogen
NOAEL: no observed adverse effect level
SD rat: Sprague-Dawley rat
KTR: Korea Testing & Research Institute
S.D: standard deviation
MFDS: Ministry of Food and Drug Safety
AVMA: American Veterinary Medical Association
IACUC: Institutional Animal Care and Use Committee
HPLC: high-performance liquid chromatography
PDA: photo-diode array
